# Round the Hospitals

**Published:** 1917-02-24

**Authors:** 


### ROUND THE HOSPITALS.

Lord Devonport's statement issued on 3rd instant calling for a curtailment of the consumption of bread, meat, and sugar by voluntary action is one which every hospital matron must keep before her and have well in mind. It is in contemplation, we understand, for the Food Controller to issue a letter of explanation to the hospitals throughout the country. This will probably emphasise the importance of hospital authorities, secretaries, stewards, and matrons realising that the consumption of meat, bread, and sugar will necessarily vary according to age, sex, occupation, and other conditions. Especially will this be so in the case of bread. The Controller's scale of consumption for households was based on a broad average to render it simpler to make a distribution amongst adults and children of both sexes and various ages. We are glad to know that the Controller recognises that in institutions the actual scale enforced must vary in some details, and that it will require modification and adjustment according to circumstances. Hospital patients must necessarily vary in respect of diets, for with them the diet often represents an important part of their treatment. We are confident that all hospital officials, especially the matrons and others more immediately concerned with the provision, cooking, and supply of food, will study these questions. Then with the aid of the dietitian or a member of the medical staff, or together, they will go carefully into the existing dietary for the patients, as well as for the staff, with the object of causing the scale for the latter to accord with Lord Devonport's estimate. It is possible, too, that, by consent, they may be able to make such modifications in the existing consumption of staple commodities by patients, as may lead to the maximum reduction possible in the circumstances of each case.

---

Last week we mentioned the splendid opportunity every matron and lady superintendent of nursing has to help her nurses to help themselves by placing their names upon the Register of the Royal British College of Nursing. It may encourage some matrons, anxious to render personal service in this matter, to be reminded, that the office organisation of the College may now be regarded as settled down to steady, continuous work. This being so, Miss Rundle, the secretary, is freer for outside duties, and enabled when invited to visit military, territorial, and Red Cross hospitals, in addition to the larger voluntary civil hospitals, to explain the working of the College, to answer questions, and to help the nurses to fill up their application forms and complete them promptly with the minimum of trouble and difficulty. The present is the time for everyone genuinely interested in the success of the College to do their utmost to make the College known and understood by nurses generally, and to increase the numbers on the College Register to at least 30,000. Every day's post brings a steady and increasing volume of applicants for registration. We hope, therefore, that Miss Rundle may have a number of invitations at the College of Nursing, 6 Vere Street, Cavendish Square, London, W., to come and help the matrons to help the nurses to help themselves.

---

The notice which we gave last week of Mr. Peter Reid, the father of the Stock Exchange, and founder of the Hospital Convalescent Home at Parkwood, Swanley, Kent, which he endowed, has proved interesting and helpful to his co-workers, we are glad to hear. One matron, in thanking us for the article, writes: "It exactly describes the man. Mr. Reid was very good to nurses and took a keen interest in the welfare of all those he employed. He was a very dear friend to his friends and I am proud to be able to say that I was one of that number." We may add that after a memorial service in the chapel at Parkwood, Mr. Peter Reid was buried on the 13th inst. in the little churchyard of St. Paul's there.

---

There is a wise rule at some of the larger hospitals that selected candidates for a vacant secretaryship, when called before the committee, shall be asked to read a page of the minutes. It may help matrons generally, and it will certainly interest all sisters who aspire to be matrons, to consider this question which a correspondent puts to us: How many hospitals employ matrons who are excellent in many ways, but who are not at their best when reading their report at the meetings of the Weekly Board or House Committee? It may be that the reader is shy or nervous or timid at being in a minority. Matrons are probably unaware that it is a practice of some hospital committees to call on their secretaries, after the matron has left the room, to repeat the substance of what she has said. The secretary's difficulty is sometimes increased in the case of a matron who introduces new subjects which are not on the agenda and of which no notice has been given. Again, it may be a useful hint for matrons to bear in mind that hospital committees are occasionally drawn from the ranks of elderly gentlemen, whose hearing is defective. The proportion of matrons who are nervous or fail to speak up, one correspondent declares, is about six to one. "That is to say, one matron in six speaks like an actress, though far be it from the writer to suggest that every successful matron must not be an actress, especially on board days." A wag has conceived the idea that after the war the staff of The Hospital might perhaps compile a shilling book on the subject of deportment. Our answer must be that if the task is ever attempted, it must include deportment for secretaries as well as for matrons when reading a report or minutes to a committee. Then possibly the secretary or matron might secretly purchase the work and send it in a plain envelope to the matron or secretary respectively of their own hospital.

**ROUND THE HOSPITALS** (*continued*).

The Royal British College of Nursing has produced a stir throughout all sections of the nursing world. Coming as it does after, or in conjunction with the war awakening, evidence is forthcoming that the general effect of this stir upon the whole nursing body, if it is utilised by matrons and sisters, may be to produce a much higher standard and apprehension in the minds of nurses now in course of their training. There is room, no doubt, in this country for a few good books dealing with nursing, especially when associated with a standard of higher intelligence and improved methods. Some matrons, to their credit, include in their course of lectures to probationers at least one upon ethics. It is now pretty general to make a strong point, and properly to impress upon each nurse, that silence is golden. No woman with a tongue like a rattle can ever become a good nurse. One test of education is that a properly trained nurse does not rattle about her patients to her friends, and certainly not about one patient to another. Unfortunately, the rattle type is far too general, and the matron or sister who first makes an absolute stand against the woman obsessed by this malady, by requesting her withdrawal from a ward or dismissal from the training school or from the staff, if she has attained full training, will win for herself a deservedly high reputation. The latest specimen of the rattle type emanates from Wales, where she is reported to have advised her patient not to remain in the institution but to seek better treatment elsewhere, and to have declared to others that the food was not good enough for them, and so induced them to refuse further treatment and demand their discharge. We should welcome the experience and views of matrons and sisters on these impediments to sound nurse-training. Good service would be done by any one of them who would write a terse account of her experience and the actual extent to which she has found the rattle type present in British institutions for the sick or attached to nursing organisations and homes within the United Kingdom?

